# RNA-Seq and WGCNA Identify Key Regulatory Modules and Genes Associated with Water-Holding Capacity and Tenderness in Sheep

**DOI:** 10.3390/ani15111569

**Published:** 2025-05-27

**Authors:** Liming Zhao, Fadi Li, Xiaoxue Zhang, Huibin Tian, Zongwu Ma, Xiaobin Yang, Qi Zhang, Mengru Pu, Peiliang Cao, Deyin Zhang, Yukun Zhang, Yuan Zhao, Jiangbo Cheng, Quanzhong Xu, Dan Xu, Xiaolong Li, Weimin Wang

**Affiliations:** 1 State Key Laboratory of Herbage Improvement and Grassland Agro-Ecosystems, Key Laboratory of Grassland Livestock Industry Innovation, Ministry of Agriculture and Rural Affairs, Engineering Research Center of Grassland Industry, Ministry of Education, College of Pastoral Agriculture Science and Technology, Lanzhou University, Lanzhou 730020, China; zlmfxy1807285865@163.com (L.Z.); lifd@lzu.edu.cn (F.L.); tianhb@lzu.edu.cn (H.T.); gsaumzw980406@163.com (Z.M.); yangxb0902@163.com (X.Y.); 13040547928@163.com (Q.Z.); pumr2023@lzu.edu.cn (M.P.); zdy1213@163.com (D.Z.); 120220900701@lzu.edu.cn (Y.Z.); zhaoyuan_10@163.com (Y.Z.); 15117098920@163.com (J.C.); 18147121406@163.com (Q.X.); 15045093462@163.com (D.X.); lixllil@163.com (X.L.); 2 College of Animal Science and Technology, Gansu Agricultural University, Lanzhou 730070, China; zhangxx@gsau.edu.cn (X.Z.); c3264887984@outlook.com (P.C.)

**Keywords:** RNA-seq, WGCNA, Hu sheep, meat quality traits, WHC, tenderness

## Abstract

Meat quality traits, particularly WHC and tenderness, are pivotal for consumer satisfaction and economic value in the sheep industry. However, their genetic regulatory mechanisms remain unclear. We used RNA-Seq and WGCNA to identify genes regulating WHC and tenderness. Sixty longissimus thoracis samples were classified into high/low WHC (HWHC vs. LWHC) and high/low tenderness (HTN vs. LTN) groups. Comparative transcriptomics identified 270 differentially expressed genes (DEGs) linked to WHC, enriched in pathways like the regulation of the ATP metabolic process and the inhibition of canonical Wnt signaling. Key DEGs (e.g., *SORBS1*, *FOXO1*, *PDE4B*, *CDH1*) correlated significantly with WHC-associated traits. For tenderness, 165 DEGs were identified, including *LEP*, *FABP4*, *PLIN1*, and *GLP1R*, enriched in PPAR signaling, fat cell differentiation, and cAMP signaling pathways. WGCNA revealed modules associated with WHC and tenderness, with hub genes (*ATP2C1*, *GSKIP*, *PATL1*, *PPARA*, *CYLD*) involved in ATP metabolism, lipid biosynthesis, and myofibril assembly. Tissue-specific gene integration prioritized muscle-enriched candidates (*METTL21C* and *ACTC1*) with strong trait correlations. Our findings unveil interconnected gene networks governing WHC and tenderness, highlighting some candidate genes as potential biomarkers for precision breeding. This study provides novel insights into the molecular determinants of meat quality, offering actionable targets to enhance mutton production sustainability and consumer appeal.

## Simple Summary

This study investigates the genetic and molecular mechanisms behind key meat quality traits in sheep, focusing on water-holding capacity (WHC) and tenderness, which are crucial for consumer satisfaction and the economic profitability of the sheep industry. The aim was to identify genes and biological pathways involved in these traits. Using RNA sequencing and gene co-expression network analysis, this study analyzed 60 samples of sheep muscle and found several genes (e.g., *FABP4*, *PLIN1*, *CYLD*) associated with WHC and tenderness. The results indicated that specific genes are associated with important pathways related to energy metabolism, fat storage, and muscle structure. These findings provide valuable insights into how meat quality is determined at the genetic level, offering potential targets for improving sheep breeding practices. The identified genes could be used to develop more efficient breeding strategies, ultimately helping to enhance meat production and meet consumer demand for higher-quality mutton.

## 1. Introduction

Mutton is a key component of meat consumption and an essential protein source for humans. As living standards rise, there is an increasing demand for high-quality meat to address the sustainability challenges associated with meat production and consumption. Meat quality is assessed through various indicators, including water-holding capacity (WHC), drip loss, cooked meat yield, intramuscular fat (IMF), shear force, and meat color. As economically critical traits in livestock production, these quality indicators demonstrate low-to-moderate genetic heritability (h^2^) [1,2,3,4]. WHC and tenderness are critical determinants of consumer acceptance and economic value in the livestock industry. WHC governs juiciness and processing yield, while tenderness directly impacts palatability, with both traits influenced by intricate interactions between post-mortem biochemical processes, muscle fiber composition, and intramuscular fat deposition [5,6]. Drip loss is a key indicator used to evaluate WHC, defined as the rate of fresh meat weight loss due to gravity at 0–4 °C over a 24 h period [7]. Previous studies on ruminants have shown that extremely low WHC resulting from myoprotein degradation is a primary factor contributing to pale, soft, and exudative (PSE) meat. In contrast, high WHC associated with elevated pH levels is linked to the development of dark, firm, and dry (DFD) meat [8]. Shear force is a useful parameter for assessing the tenderness, juiciness, and other qualities of cooked meat, offering a direct indication of meat tenderness [9]. Within a specific range, lower shear force values are indicative of more tender meat [10].

Meat quality traits are affected by various factors, including environmental conditions, dietary management, and, particularly, genetic factors [11]. In sheep, genetic and molecular mechanisms underlying these traits remain poorly characterized, despite their significance for breeding programs aiming to enhance meat quality. Considerable efforts have been made to identify genetic determinants underlying the phenotypic diversity of meat-related traits in various populations. Several key pathways, including lipid and fatty acid binding, the adipocytokine signaling pathway, PPAR signaling pathway [12], AMPK signaling pathway [13], and arachidonic acid metabolism [14], have been linked to meat quality traits in sheep. Transcriptomics have enabled the systematic exploration of gene regulatory networks associated with meat quality. Zhang et al. [15] conducted a comparative analysis between two pig breeds to identify genetic and metabolic factors affecting meat quality. They discovered several functional genes linked to meat quality traits, including *PPP1R3B*, *PPARGC1A*, and *SOCS1*. Zhao et al. [16] measured meat quality traits, including drip loss, in 28 Duroc pigs and performed a comparative transcriptomic analysis on individuals exhibiting significantly low and high levels of this trait. They identified key genes linked to drip loss, including *TNC*, *ITGA5*, *ITGA11*, *THBS3*, and *CD44*. Complementarily, weighted gene co-expression network analysis (WGCNA) provides a powerful framework to delineate the modules of co-regulated genes functionally associated with meat quality traits in sheep. Several key genes were identified, such as *P4HA2*, *FBXL4* [13], *GLB1*, *PLD3*, *LPCAT2* [17], *BUB1*, *SKA1*, and *PLA2G5* [18].

In this study, we hypothesize that genetics is an important factor causing differences in sheep meat quality. We employed RNA-Seq and WGCNA on phenotypically stratified Hu sheep cohorts to systematically identify key regulatory modules and potential genes associated with meat quality traits. The primary aim of this study was to discover valuable genes and pathways related to meat water-holding capacity and tenderness in sheep and to provide new insights into differences in meat quality, laying a theoretical foundation for breeding sheep with improved meat quality. The findings advance our understanding of molecular determinants underlying critical meat quality traits while providing actionable targets for marker-assisted selection in sheep breeding programs.

## 2. Materials and Methods

### 2.1. Experimental Design and Tissue Sample Collections

**Figure 1:**
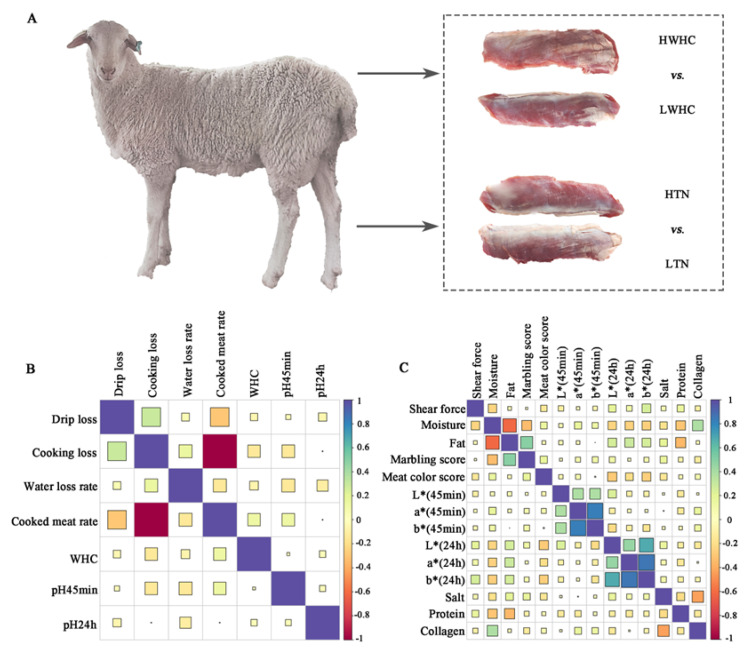
Analysis of the relationship between meat quality traits. (**A**) The experimental design of this study. (**B**) The correlation analysis between WHC-related traits. (**C**) The correlation analysis between tenderness-related traits.

A group of 169 male Hu sheep was bred at Minqin Defu Agriculture Co., Ltd. (Gansu Province, China) under consistent environmental conditions from post-weaning (56 days old) to slaughter (180 days old). The experimental protocol comprised three sequential phases: 14-day environmental adaptation, 10-day pre-test conditioning, and a 100-day formal trial period. The sheep underwent a 12 h fast before slaughter. Immediately post-slaughter, a portion of the longissimus thoracis (LT) samples was collected for meat quality analysis, while duplicate samples were frozen in liquid nitrogen and stored at −80 °C for subsequent analysis. According to meat quality traits, sixty healthy and similarly weighted (approximately 45 kg) sheep were categorized into two comparisons (Figure 1A): (1) different meat water-holding capacity: HWHC (high water-holding capacity, *n* = 15, WHC = 0.7899 ± 0.007), LWHC (low water-holding capacity, *n* = 15, WHC = 0.7391 ± 0.006); (2) different meat tenderness: HTN (high tenderness, *n* = 15, shear force = 57.34 ± 2.52), LTN (low tenderness, *n* = 15, shear force = 75.36 ± 1.11).

### 2.2. Meat Quality Assessment

#### 2.2.1. Determining Water-Holding Capacity in Meat

The WHC was measured by the water-holding capacity tester (RH-1000, Runhu Instrument Co., Ltd., Guangzhou, China). Drip loss was calculated using the equation (Δm/m_0_) × 100, where Δm corresponds to the mass loss during dripping and m_0_ represents the pre-drip sample mass. Cooking loss was assessed by precisely weighing meat samples (30 ± 1 g), which were vacuum sealed in self-sealing bags and heated in an 80 °C water bath for 45 min. Post-heating, samples were blotted dry and equilibrated to ambient temperature prior to reweighing. Cooking loss (%) was defined as the percentage mass reduction relative to the initial weight. Using a portable pH meter (testo), the pH of the muscle tissue was evaluated 45 min and 24 h after slaughter, with the samples refrigerated between 0 and 4 °C throughout the 24 h storage period.

#### 2.2.2. Analysis of Tenderness in Meat

To assess meat tenderness, a Warner–Bratzler shear device (GR Manufacturing, Manhattan, KS, USA) was employed to determine the shear force value. Six random subsections were extracted from the meat samples using a 1.27 cm diameter circular core sampler, ensuring alignment with the direction of the muscle fibers. The muscle’s chemical composition, including fat, moisture, salt, protein, and collagen, was assessed using a FoodScan Meat Analyzer (FOSS ANALYTICAL A/S, Hillerød, Danmark). The degree of marbling was assessed using a 0–5 scoring system, with 5 indicating maximal intramuscular fat content. The color characteristics of the muscle, including L*, a*, and b* values, were evaluated with a colorimeter (KONICA MINOLTA, Konica Minolta, Japan) at 45 min and 24 h after slaughter, where L* indicates brightness, a* signifies redness, and b* corresponds to yellowness. The meat color score was assessed visually using a colorimetric card, with scores ranging from 0 to 5, where higher values indicate a darker color.

### 2.3. RNA Extraction, Library Preparation, and Transcriptome Sequencing

To isolate RNA, longissimus thoracis tissue samples were processed using the TransZol Kit (TransGen Biotech, Beijing, China), adhering to the manufacturer’s recommended procedure. The integrity and concentration of extracted RNA were subsequently evaluated through the RNA Nano 6000 Assay Kit, employing the Agilent Bioanalyzer 2100 system (Agilent Technologies, Santa Clara, CA, USA). Then, high-quality RNA samples with an OD 260/280 ratio greater than 1.8 and an RNA Integrity Number (RIN) above 9 were utilized to construct cDNA libraries for RNA sequencing (Table S1). For cDNA library preparation, the NEBNext^®^ UltraTM RNA Library Prep Kit designed for Illumina^®^ (NEB, Ipswich, MA, USA) was employed, following the manufacturer’s guidelines closely. The initial step involved mRNA enrichment from total RNA through poly-T oligonucleotide-conjugated magnetic beads. First-strand cDNA synthesis was catalyzed by M-MuLV Reverse Transcriptase with random hexamers as primers. Subsequently, second-strand cDNA was produced through the combined action of DNA Polymerase I and RNase H. To ensure the acquisition of optimal cDNA fragments, the library was processed with the AMPure XP system for purification. The final cDNA libraries were subjected to sequencing on the Illumina Novaseq platform, generating 150 bp paired-end reads for downstream analysis.

### 2.4. RNA Sequencing Data Analysis

To ensure data quality, raw sequencing reads were processed to remove adapter-containing sequences, poly-N segments, and low-quality reads, yielding high-quality clean data for downstream analysis. The purified reads were aligned to the ovine reference genome assembly Oar_rambouillet_v1.0 using Hisat2 (version 2.2.1). Gene expression levels were quantified and normalized through the transcripts per million (TPM) method, implemented via computational scripts. For comparative transcriptomic analysis, the DESeq2 package (version 1.44.0) was employed to evaluate differential gene expression between experimental groups, utilizing a negative binomial distribution model. Genes meeting the stringent criteria of *p*-value < 0.05 and fold changes > 2 were classified as significantly differentially expressed genes (DEGs).

### 2.5. Weighted Gene Co-Expression Network Analysis (WGCNA)

WGCNA was conducted using the WGCNA package (version 1.73) [19] in R, employing the aggregated expression matrix derived from longissimus thoracis tissue samples. A stringent gene selection criterion was applied to filter out lowly expressed genes and those with small variability. Genes with a cumulative read count > 10 across all samples were retained, and the top 75% of genes were selected based on their median absolute deviation (MAD), while genes with a MAD < 0.01 were removed in each sample. The scale-free topology criterion (R^2^ value > 0.85) was applied to determine the optimal soft thresholding power (β = 1–30), ensuring that the networks displayed an approximately scale-free topology. Network construction involved the generation of a topological overlap matrix (TOM) derived from correlation expression values, which served as the basis for gene hierarchical clustering. To establish functional correlations, phenotypic traits related to meat water-holding capacity and tenderness were included in the module–trait relationship analysis, respectively. The statistical significance of module–trait associations was determined at thresholds of *p* < 0.05 (*) and *p* < 0.01 (**). Network visualization and analysis were performed using Cytoscape (version 3.10.0) to elucidate the architecture of co-expressed gene regulatory networks.

### 2.6. Functional Enrichment Analysis of Genes

The biological significance of the identified gene set was assessed through a functional enrichment analysis using the clusterProfiler [20] R package (v4.6.0). The analysis encompassed both Gene Ontology (GO, http://www.geneontology.org, accessed on 1 May 2025) categories and Kyoto Encyclopedia of Genes and Genomes (KEGG, http://www.kegg.jp, accessed on 1 May 2025) pathways to provide comprehensive insights into the biological functions and molecular pathways associated with the gene list. GO terms and KEGG pathways with a *p*-value of less than 0.05 were regarded as significantly enriched.

### 2.7. Investigation of Muscle Tissue-Specific Genes

Building upon our extensive transcriptomic atlas [21] derived from RNA-Seq analysis across ten distinct ovine tissues (including muscle, liver, testis, lung, kidney, jejunum, rumen, lymph, tail fat, and hypothalamus), we conducted an integrative analysis to identify overlapping genes between muscle tissue-specific genes (TSGs) and differentially expressed genes across both up-regulated and down-regulated profiles. In brief, we first identified the TSGs of muscle based on RNA-Seq data from ten tissues (with expression levels more than three times higher than those in other tissues) and then performed a Venn analysis between the muscle-specific genes and the DEGs identified in this study. This systematic approach enabled the identification and prioritization of potential molecular markers associated with critical meat quality attributes in sheep.

### 2.8. qRT-PCR Analysis

To validate RNA-Seq results, qRT-PCR was performed using the SYBR Premix Ex Taq™ kit (Takara Biotechnology, Shiga, Japan) on a LightCycler 480 (Roche Applied Science, Mannheim, Germany). Total RNA was extracted from LT tissues for qPCR, as described previously. The reaction mixture was 20 µL, consisting of 2 μL of cDNA, 0.8 μL of each primer, 10 μL of 2 × SYBR Green PCR Master Mix, and 6.4 μL of RNase-free water. The qPCR conditions were set as follows: initial denaturation at 95 °C for 3 min, followed by 40 cycles of 15 s at 95 °C and 20 s at 72 °C. Primers were designed with the Oligo 7.0 software, and the detailed information is listed in Table 1. The *ACTB* gene served as the internal control for normalizing the expression of the target gene. The relative expression level was calculated by the 2^−ΔΔCT^ method [22].

## 3. Results

### 3.1. Correlation Analysis of Meat Quality Traits

To elucidate the intrinsic relationships between meat water-holding and tenderness-related attributes, we performed a comprehensive correlation analysis of twenty-one key meat quality traits. The heatmap visualization revealed distinct correlation patterns, with WHC demonstrating negative correlations with drip loss (r = −0.05), cooking loss (r = −0.13), and the water loss rate (r = −0.06), while showing a positive association with the cooked meat rate (r = 0.13). Notably, both early (pH 45 min) and ultimate (pH 24 h) pH values exhibited negative correlations with drip loss (r = −0.02 and −0.06, respectively) and the water loss rate (r = −0.14 and −0.11, respectively), aligning with the established biochemical principles of pH-dependent protein denaturation and water retention efficiency (Figure 1B). Figure 1C illustrates the relationships between shear force and key meat quality parameters, including fat content, moisture content, marbling score, and colorimetric attributes (L*, a*, and b* values and meat color score). Notably, lower shear force values, indicative of greater tenderness, are associated with higher moisture content (r = −0.25), fat content (r = −0.05), meat color score (r = −0.14), and elevated marbling scores (r = −0.02), suggesting that these factors play a pivotal role in enhancing meat tenderness. Conversely, meat samples exhibiting higher shear force values were predominantly characterized by reduced moisture content and lower marbling scores.

### 3.2. Comparison of Meat Quality Traits Among Different Groups

**Figure 2:**
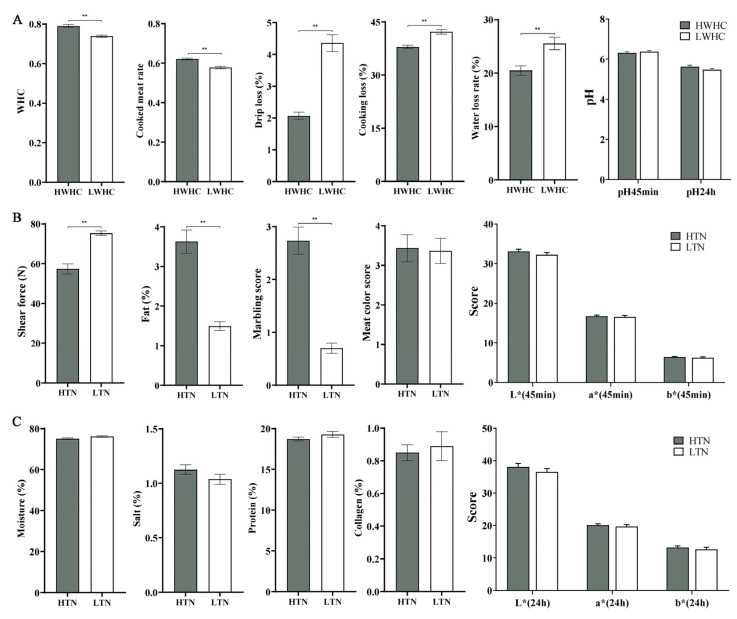
Comparative analysis of meat quality traits between different comparisons. (**A**) Comparison of WHC-related traits between the HWHC and LWHC groups, including cooked meat rate, drip loss, cooking loss, water loss rate, and pH. (**B**,**C**) Comparison of tenderness-related traits between the HTN and LTN groups, including shear force, fat content, marbling score, meat color score, moisture, salt, protein, collagen content, and colorimetric attributes. *Note*: double asterisks indicate extremely significant differences between the different groups (*p* < 0.01).

The meat quality traits were compared among the defined groups, including HWHC vs. LWHC and HTN vs. LTN, as illustrated in Figure 2. Figure 2A demonstrates significant differences in water-holding-related traits between the HWHC and LWHC groups. The HWHC group exhibited a significantly higher WHC and cooked meat rate compared to the LWHC group (*p* < 0.05). Conversely, the drip loss, cooking loss, and water loss rate were significantly lower in the HWHC group (*p* < 0.05). However, no significant differences were observed in pH values at 45 min (pH 45 min) and 24 h (pH 24 h) post-mortem between the two groups (*p* > 0.05). Figure 2B,C highlight the comparison of tenderness-related traits between the HTN and LTN groups. The HTN group exhibited markedly reduced shear force (*p* < 0.05), indicating greater tenderness, as well as a significantly higher fat content and marbling score compared to the LTN group (*p* < 0.05). Other traits, including moisture, meat color score, colorimetric attributes (L*, a*, and b* at 45 min and 24 h), salt, protein, and collagen content, showed no significant variation between the two groups (*p* > 0.05).

### 3.3. Identification of DEGs Associated with Meat Water-Holding Capacity

**Figure 3:**
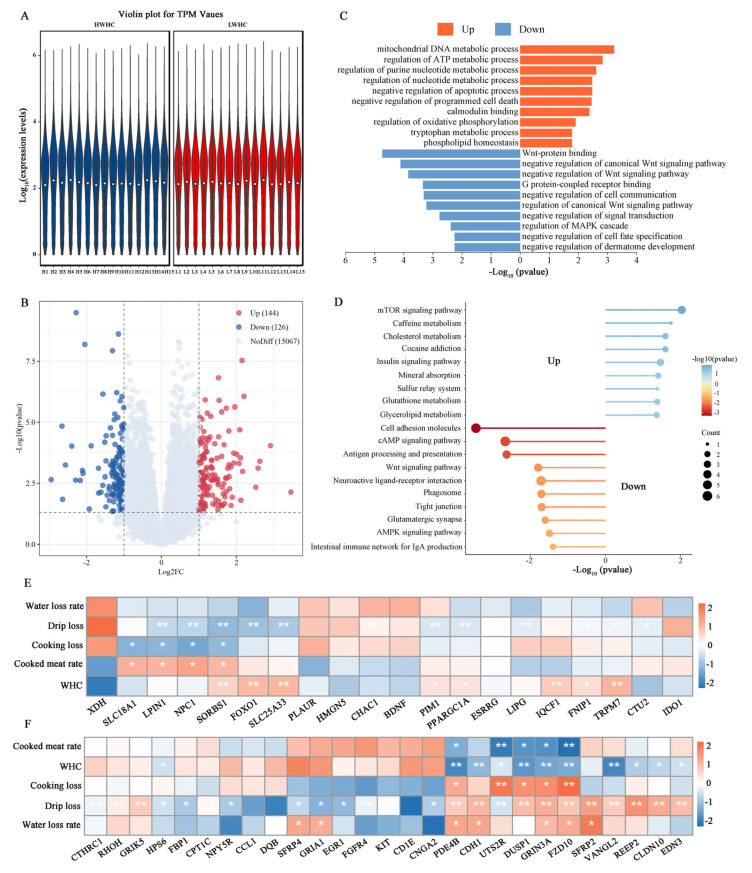
Comparative transcriptome analysis between the HWHC and LWHC groups. (**A**) Distribution of gene expression levels in the HWHC and LWHC groups. (**B**) Volcano plot in the HWHC vs. LWHC comparison. (**C**) GO enrichment analysis of DEGs in the HWHC vs. LWHC comparison. (**D**) KEGG analysis of DEGs in the HWHC vs. LWHC comparison. (**E**) Correlation analysis between up-regulation of DEGs and WHC-related traits. (**F**) Correlation analysis between down-regulation of DEGs and WHC-related traits. *Note*: asterisks indicate significant differences (*p* < 0.05), double asterisks indicate extremely significant differences (*p* < 0.01).

We obtained a total of 141,290,130,2 raw reads in the WHC group after Illumina sequencing. After adaptor removal and filtering out low-quality sequences, a total of 139,040,211,6 high-quality clean reads were generated. The Q20 and Q30 percentages ranged from 98.72% to 98.92% and 96.31% to 96.84%, respectively (Table S1). To elucidate the molecular mechanisms underlying meat water-holding capacity, a comparative transcriptomic analysis was conducted between the HWHC and LWHC groups. Figure 3A illustrates the distribution of gene expression levels across biological replicates, demonstrating consistent intra-group reproducibility. Figure 3B presents a volcano plot of differentially expressed genes, identifying a total of 270 DEGs (*p* < 0.05, fold change > 2), including 144 up-regulated and 126 down-regulated genes in the HWHC group compared to the LWHC group (Table S2). Functional enrichment analysis revealed distinct biological processes and pathways associated with these DEGs. Figure 3C shows GO enrichment results, where up-regulated DEGs were significantly enriched in processes such as “smooth muscle contractile fiber”, “positive regulation of cell growth”, “lipoprotein lipase activity”, “regulation of ATP metabolic process”, “regulation of generation of precursor metabolites and energy”, and “positive regulation of lipoprotein particle clearance”, while down-regulated DEGs were primarily concentrated in the “regulation of Wnt signaling pathway”, “canonical Wnt signaling pathway”, “regulation of MAP kinase activity”, “tissue development”, and “Wnt signaling pathway” terms.

Figure 3D highlights KEGG pathway analysis, identifying up-regulated DEGs in pathways including the “mTOR signaling pathway”, “Glutathione metabolism”, “Insulin signaling pathway”, and “Mineral absorption”, whereas down-regulated DEGs were enriched in “Cell adhesion molecules”, “cAMP signaling pathway”, and “Wnt signaling pathway” pathways. Furthermore, a correlation analysis between key pathway genes and meat quality traits was performed. Up-regulated DEGs, such as *SLC18A1* (FC = 3.13), *LPIN1* (FC = 2.29), *NPC1* (FC = 2.54), and *FOXO1* (FC = 2.11), exhibited significant positive correlations with the WHC and cooked meat rate, and negative correlations with drip loss and cooking loss (Figure 3E). Conversely, down-regulated DEGs, including *PDE4B* (FC = 0.40), *CDH1* (FC = 0.46), *UTS2R* (FC = 0.35), *DUSP1* (FC = 0.48), *GRIN3A* (FC = 0.34), and *FZD10* (FC = 0.45), showed positive correlations with cooking loss, drip loss, and the water loss rate but negative correlations with the WHC and cooked meat rate (Figure 3F).

### 3.4. Identification of DEGs Associated with Meat Tenderness

**Figure 4:**
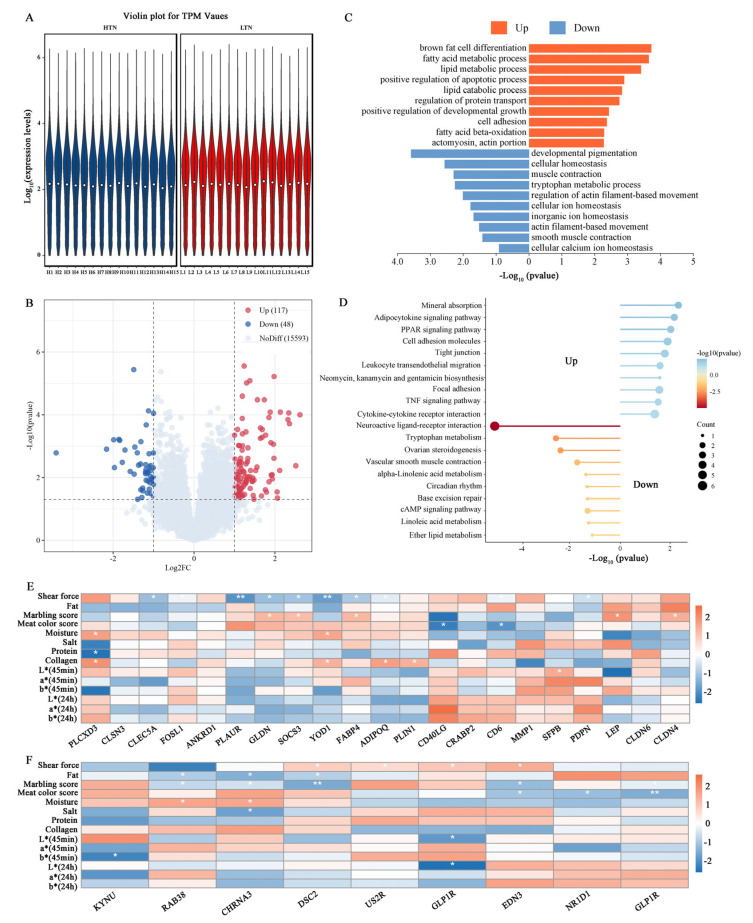
Comparative transcriptome analysis between the HTN and LTN groups. (**A**) Distribution of gene expression levels in the HTN and LTN groups. (**B**) Volcano plot in the HTN vs. LTN comparison. (**C**) GO enrichment analysis of DEGs in the HTN vs. LTN comparison. (**D**) KEGG analysis of DEGs in the HTN vs. LTN comparison. (**E**) Correlation analysis between up-regulation of DEGs and WHC-related traits. (**F**) Correlation analysis between down-regulation of DEGs and WHC-related traits. *Note*: asterisks indicate significant differences (*p* < 0.05), double asterisks indicate extremely significant differences (*p* < 0.01).

In the meat tenderness group, a total of 1,356,026,058 raw reads were produced. Following quality control, 1,333,674,286 clean reads were retained, with Q20 and Q30 quality values exceeding 98.72% and 96.31%, respectively (Table S1). To identify DEGs associated with meat tenderness, we performed a transcriptome comparison between the HTN and LTN groups. Gene expression distribution across biological replicates, visualized by violin plots (Figure 4A), demonstrated high reproducibility within groups. Comparative analysis identified 165 DEGs, including 117 up-regulated and 48 down-regulated genes in HTN relative to LTN (Figure 4B, Table S3). GO analysis showed that up-regulated DEGs were significantly enriched in processes related to lipid metabolism and skeletal assembly, including the “cellular lipid catabolic process”, “biological adhesion”, “cellular lipid metabolic process”, “fatty acid catabolic process”, “fat cell differentiation”, “skeletal myofibril assembly”, and “skeletal muscle thin filament assembly”. Down-regulated DEGs were predominantly linked to muscle function and energy metabolism, such as the “muscle system process”, “phasic smooth muscle contraction”, “cell-cell adherens junction”, and “energy reserve metabolic process” terms (Figure 4C).

KEGG pathway analysis (Figure 4D) further highlighted that up-regulated DEGs were enriched in the “PPAR signaling pathway”, “Adipocytokine signaling pathway”, and “AMPK signaling pathway”, while down-regulated DEGs were linked to “steroid hormone biosynthesis” and “cAMP signaling pathway”. Notably, key DEGs within these pathways exhibited significant correlations with meat quality traits. Up-regulated genes, such as *PLAUR* (FC = 2.44), *GLDN* (FC = 3.73), *SOCS3* (FC = 2.11), *YOD1* (FC = 2.15), and *FABP4* (FC = 2.44), showed negative correlations with shear force and positive associations with the marbling scores and meat color scores (Figure 4E). Conversely, down-regulated genes were positively correlated with shear force and negatively linked to the fat content and marbling score, including *DSC2* (FC = 0.40) and *EDN3* (FC = 0.39, Figure 4F).

### 3.5. Combined Analysis of TSGs and DEGs

**Figure 5:**
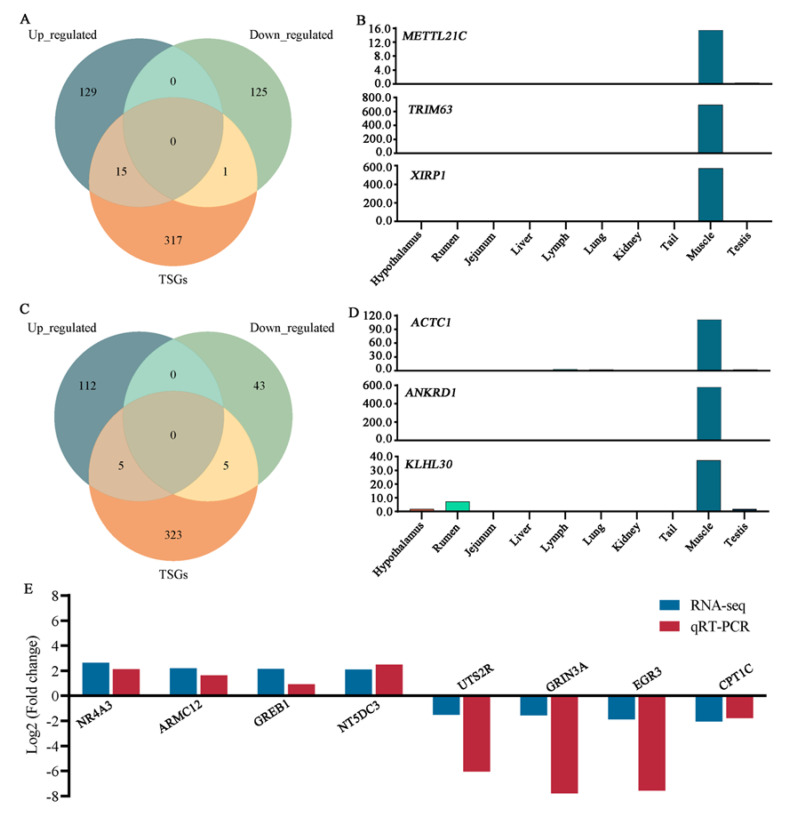
Integration analysis of TSGs and DEGs. (**A**) Venn analysis of DEGs and muscle tissue-specific genes in the HWHC vs. LWHC groups. (**B**) Expression levels of *METTL21C*, *TRIM63*, and *XIRP1* genes in 10 tissues. (**C**) Venn analysis of DEGs and muscle tissue-specific genes in the HTN vs. LTN groups. (**D**) Expression levels of *ACTC1*, *ANKRD1*, and *KLHL30* genes in 10 tissues. (**E**) RNA-Seq data validation.

In the HWHC vs. LWHC comparison, sixteen DEGs were found to be muscle tissue-specific, including fifteen up-regulated genes and one down-regulated gene (Figure 5A). For instance, the expression levels of the *METTL21C* (FC = 3.61), *TRIM63* (FC = 2.14), and *XIRP1* (FC = 2.19) genes in muscle tissue were higher compared to the other nine tissues (Figure 5B). In the comparison of the HTN and LTN groups, ten DEGs were classified as muscle tissue-specific, consisting of five up-regulated and five down-regulated genes (Figure 5C). Notably, genes such as *ACTC1* (FC = 2.52), *ANKRD1* (FC = 2.24), and *KLHL30* (FC = 2.22) exhibited higher expression levels in muscle tissue relative to the other nine tissues examined (Figure 5D).

### 3.6. Validation of RNA-Seq Data Using qRT-PCR

To verify the RNA-seq data, eight genes (*NR4A3*, *ARMC12*, *GREB1*, and *NT5DC3*, up-regulation genes; *UTS2R*, *GRIN3A*, *EGR3*, and *CPT1C,* down-regulation genes) were randomly chosen and validated using qRT-PCR. The qRT-PCR results aligned with the RNA-seq data (Figure 5E), confirming the reliability of the sequencing results.

### 3.7. Gene Co-Expression Modules Related to Meat Quality Traits in Sheep

#### 3.7.1. Identification of Hub Genes Associated with Meat Water-Holding Capacity in Sheep

**Figure 6:**
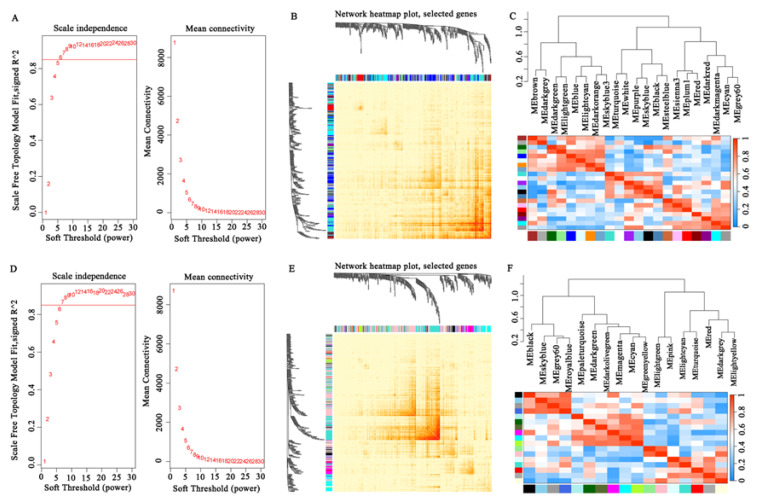
(**A**,**D**) Selection of soft threshold power for traits related to WHC and tenderness. (**B**,**E**) Cluster analysis of the relationships between different modules for traits related to WHC and tenderness, the darker the color, the higher the correlation. (**C**,**F**) Module gene correlation heatmap for traits related to WHC and tenderness.

**Figure 7:**
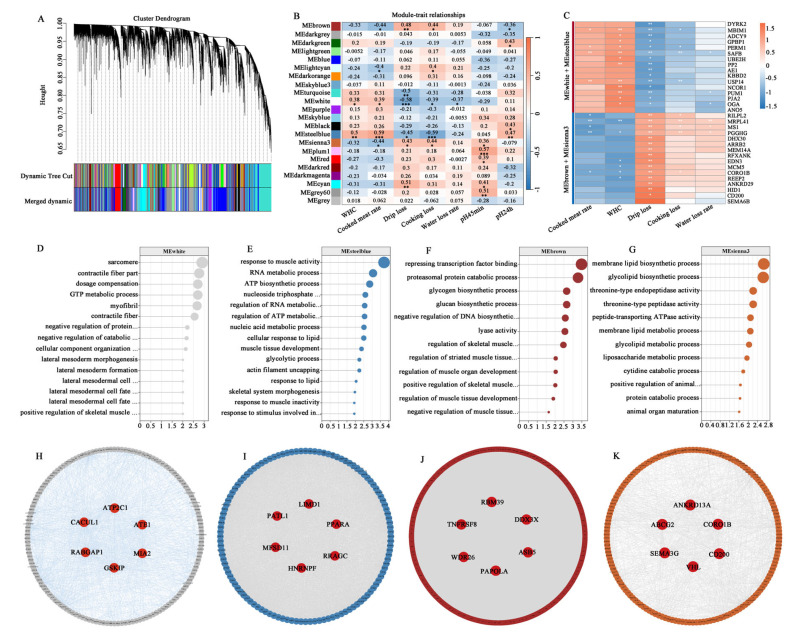
WGCNA analysis of WHC-related traits. (**A**) Gene clustering dendrogram with color-coded modules. (**B**) Relationship between the module eigengene and WHC-related traits. (**C**) The correlation between genes in key modules and WHC-related traits. (**D**–**G**) GO enrichment analysis of genes in the MEwhite, MEsteelblue, MEbrown, and MEsienna3 modules. (**H**–**K**) Interaction network of genes in the MEwhite, MEsteelblue, MEbrown, and MEsienna3 modules. *Note*: *Note*: “*” indicate significant differences (*p* < 0.05), “**” indicate extremely significant differences (*p* < 0.01), “***” indicate extremely significant differences (*p* < 0.001).

To investigate the molecular networks underlying meat water-holding capacity, WGCNA was performed. After determining an optimal soft threshold of 6 (Figure 6A), co-expression modules were constructed, revealing distinct clusters of genes with shared expression patterns (Figure 7A). The hierarchical clustering patterns and co-expression network correlations among distinct modules are visually represented through dendrogram and heatmap analysis in Figure 6B,C. Among these, the MEwhite and MEsteelblue modules exhibited significant positive correlations with WHC and the cooked meat rate but negative correlations with drip loss, cooking loss, and the water loss rate. Conversely, the MEbrown and MEsienna3 modules exhibited significant positive correlations with drip loss and cooking loss, and negative correlations with the cooked meat rate (Figure 7B). Based on gene significance (GS), we then selected the top genes from these modules for correlation analysis with meat quality traits. As shown in Figure 7C, genes in the MEwhite and MEsteelblue modules were positively correlated with WHC and the cooked meat rate, including *MBIM1*, *PERM1*, *SAFB*, and *USP14*. In contrast, genes in the MEbrown and MEsienna3 modules exhibited opposing expression patterns, such as *MRPL41*, *PGGHG*, and *CORO1B*. The functional enrichment analysis of these modules highlighted critical pathways linked to WHC regulation. Genes within the MEwhite and MEsteelblue modules were predominantly involved in metabolic processes and muscle development, such as the “GTP metabolic process”, “negative regulation of catabolic process”, “ATP biosynthetic process”, “contractile fiber part”, “myofibril”, “positive regulation of skeletal muscle tissue growth”, and “skeletal system morphogenesis” (Figure 7D,E). Genes in the MEbrown and MEsienna3 modules were significantly enriched in the “regulation of skeletal muscle cell differentiation”, “regulation of striated muscle tissue development”, “negative regulation of muscle tissue development”, and “membrane lipid biosynthetic process” terms (Figure 7F,G). Gene interaction network analysis further identified central hub genes with high connectivity, including *ATP2C1*, *CACUL1*, *GSKIP*, *PATL1*, *MFSD11*, *LIMD1*, *RBM39*, *DDX3X*, *ASB5*, *ABCG2*, *SEMA3G*, and *CORO1B* (Figure 7H–K).

#### 3.7.2. Identification of Hub Genes Associated with Meat Tenderness in Sheep

**Figure 8:**
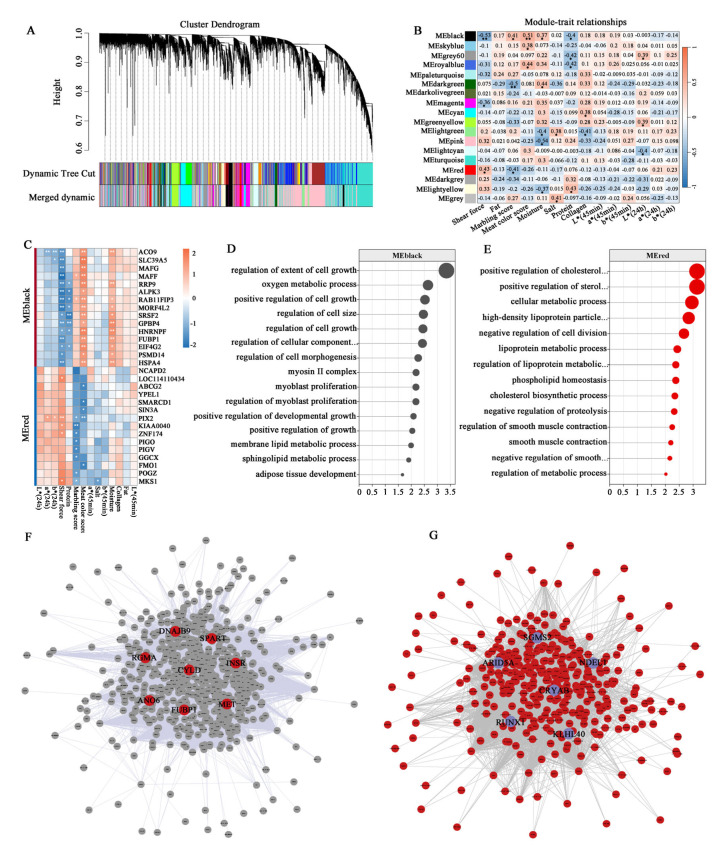
WGCNA analysis of tenderness-related traits. (**A**) Gene clustering dendrogram with color-coded modules. (**B**) Relationship between the module eigengene and tenderness-related traits. (**C**) The correlation between genes in key modules and tenderness-related traits. (**D**,**E**) GO enrichment analysis of genes in the MEblack and MEred modules. (**F**,**G**) Interaction network of genes in the MEblack and MEred modules. *Note*: asterisks indicate significant differences (*p* < 0.05), double asterisks indicate extremely significant differences (*p* < 0.01).

For meat tenderness-related traits, co-expression network construction using a soft threshold of 7 (Figure 6D) resolved distinct gene clusters (Figure 8A), with module–trait relationship analysis revealing the MEblack module as significantly negative correlated with shear force but significantly positive correlated with the marbling score, meat color score, and moisture, while the MEred module was positively correlated with shear force and negative correlated with the marbling score (Figure 8B). The top genes in the MEblack module, including *ACO9*, *SLC39A5*, *MAFG*, *RRP9*, and *ALPK3*, were negatively correlated with shear force. In contrast, the top genes in the MEred module, such as *PIX2*, *KIAA0040*, *ZNF174*, and *MKS1*, showed positive correlations with shear force and negative correlations with the marbling score and meat color score (Figure 8C). Genes in the low shear force-associated module (MEblack) were enriched in pathways including the “regulation of extent of cell growth”, “regulation of cell size”, “myoblast proliferation”, “adipose tissue development”, and “sphingolipid metabolic process” terms (Figure 8D). The MEred module genes were linked to the “positive regulation of cholesterol metabolic process”, “negative regulation of cell division”, “regulation of lipoprotein metabolic process”, “negative regulation of smooth muscle contraction”, and “regulation of metabolic process” terms (Figure 8E). A topological analysis of the gene interaction network identified *CYLD*, *SPART*, *INSR*, *ANO6*, *CRYAB*, *ARID5A*, *SGMS2*, and *KLHL40* as hubs with maximal connectivity scores, indicating their pivotal regulatory roles (Figure 8F,G).

## 4. Discussion

As consumers become more conscious of where their meat comes from and its quality, the meat industry faces new challenges in producing high-quality meat [23]. Mutton is an essential global protein source, with consumer preferences increasingly prioritizing meat quality attributes like sensory, nutritional, and processing qualities. Among these, sensory quality is a crucial factor in shaping consumer preferences for meat products [24,25]. Tenderness and juiciness are the main sensory characteristics of meat [26] that significantly impact palatability and processing efficiency, ultimately influencing the market value and sustainability of meat production systems. This study systematically identifies key regulatory genes and pathways associated with WHC and tenderness in Hu sheep by comparing sheep with varying levels of meat WHC and tenderness and integrating RNA-Seq and WGCNA. The aim is to provide new insights for precision breeding programs. The correlation analysis between various meat quality indicators reveals that drip loss, cooking loss, and the water loss rate are negatively correlated with WHC, whereas the cooked meat rate is positively correlated with WHC, aligning with prior research findings [27,28]. The muscle consists of muscle fibers and water, with the water content playing a crucial role in determining its tenderness [29]. In this study, shear force was negatively correlated with moisture content, fat content, and the marbling score.

In the comparison between the HWHC and LWHC groups, a total of 270 DEGs (including 144 up-regulated and 126 down-regulated genes) were identified. The up-regulated DEGs were primarily enriched in processes such as “smooth muscle contractile fiber”, “positive regulation of cell growth”, “lipoprotein lipase activity”, and “regulation of ATP metabolic process”. After the tricarboxylic acid cycle stops in post-mortem muscles and creatine phosphate stores are depleted, ATP production relies only on glycolysis, which becomes insufficient [30]. At this stage, as actomyosin accumulates, the muscle gradually stiffens, and its water-holding capacity decreases [31]. Skeletal muscle generates and stores energy through the oxidation of glycogen, carbohydrates, and fats via oxidative phosphorylation. This process involves the production of ATP as electrons are transferred from NADH or FADH_2_ to O_2_ through a chain of electron carriers [32]. Therefore, the up-regulated genes may further influence meat water-holding capacity by regulating ATP metabolism. The down-regulated DEGs primarily participated in pathways like the “regulation of Wnt signaling pathway”, “canonical Wnt signaling pathway”, “regulation of MAP kinase activity”, and “Wnt signaling pathway”. The Wnt signaling pathway is commonly recognized as a key activator of myogenesis, working in conjunction with myogenic regulatory factors (MRFs) [33]. Therefore, we hypothesize that the down-regulated DEGs may affect myogenesis by negatively regulating the Wnt pathway, thereby leading to differences in meat water-holding capacity. We identified several DEGs significantly associated with WHC, such as *SORBS1*, *FOXO1*, *SLC25A33*, and *TRPM7* (up-regulated DEGs, positively correlated with water-holding capacity), and *PDE4B*, *CDH1*, *UTS2R*, *DUSP1*, *GRIN3A*, and *FZD10* (down-regulated DEGs, negatively correlated with water-holding capacity). The *SORBS1* gene is a potential key factor linked to an increased IMF content in cattle [34]. The FOXO transcription factors play a key role in various essential bodily functions [29]. It has been reported that *FOXO1* is crucial for skeletal muscle type determination and inhibits the formation of MyHC I [35]. Additionally, Won et al. [36] identified, through GWAS analysis, that *TRPM7* is associated with the total collagen content in beef, which can influence meat tenderness. Among the down-regulated genes, *PDE4B* has been reported to be associated with the pH24 of pork [37], *CDH1* with the number of muscle satellite cells in animals [38], and *UTS2R* with the fatty acid composition in cattle [39]. Therefore, these genes and pathways could be essential in regulating the water-holding capacity of meat.

In the HTN vs. LTN comparison, 165 DEGs were detected, comprising 117 up-regulated and 48 down-regulated genes. For the up-regulated DEGs (including *LEP*, *FABP4*, *PLIN1*, *ADIPOQ*), several pathways associated with adipogenesis were significantly enriched, such as the “cellular lipid catabolic process”, “fatty acid catabolic process”, “PPAR signaling pathway”, and “fat cell differentiation”. The PPAR signaling pathway plays a key role in regulating carbohydrate and lipid metabolism, along with muscle development and growth [40]. The content of intramuscular fat (IMF) is a key factor in determining meat’s tenderness, flavor, and juiciness, all of which are crucial for evaluating its potential eating quality [41,42]. In 1994, Zhang first identified the leptin (LEP) gene [43]. This gene is a key candidate for important economic traits in livestock and plays a role in reproduction, immunity, growth, metabolism, and fat deposition in animals [44,45]. *FABP4* is essential for binding and transporting long-chain fatty acids in mammals [46]. It has been reported that variations in the *FABP4* gene are linked to growth traits [47], meat quality traits, and carcass traits [48] in sheep. The phosphorylation of *PLIN1* is crucial in regulating fat metabolism, influencing both lipolysis and fat storage in adipocytes [49]. The down-regulated genes are primarily enriched in the pathways of the muscle system process, the regulation of striated muscle contraction (*DSC2*), vascular smooth muscle contraction (*EDN3*), and the cAMP signaling pathway (*GLP1R*). cAMP signaling can enhance lipid metabolism and differentiation [50,51]. Studies have shown that GLP-1R agonists facilitate the browning of white adipose tissue, which may contribute to their weight loss effects [52].

WGCNA provided a systems-level perspective, identifying co-expression modules functionally linked to WHC and tenderness. The choice of threshold is crucial, as it influences both the network structure and the biological interpretation. An excessively low R^2^ value can result in an unreliable network. In this study, the optimal soft threshold powers β for the WHC and tenderness groups were determined to be 6 and 7, respectively, to meet the requirement of a scale-free topology index R^2^ exceeding 0.85. The pathways enriched in the gene sets of the MEwhite and MEsteelblue modules emphasize the role of GTP/ATP metabolism and myofibril assembly in water retention, likely maintaining osmotic balance and sarcomere integrity post-mortem. Several hub genes, such as *ATP2C1*, have been reported to be associated with backfat thickness in pigs [53]; variations in *GSKIP* can affect carcass and growth traits in sheep [54]; *PATL1* is linked to carcass and meat quality traits in pigs [55], while *PPARA* is associated with IMF content in pigs [56]. These genes may play a crucial role in the water-holding capacity of sheep meat. For tenderness, the MEblack module (*CYLD*) highlights adipogenesis as a critical regulator, including the membrane lipid metabolic process and the adipose tissue development pathway, which are significantly enriched. Studies have reported that the *CYLD* gene is significantly associated with meat quality traits in cattle [57]. These network-driven discoveries extend beyond conventional DEG analysis, revealing interconnected gene clusters that collectively shape meat quality phenotypes. The integration of tissue-specific genes (TSGs) with DEGs prioritized candidates such as *METTL21C* and *ACTC1*, which exhibit muscle-enriched expression and strong correlations with target traits. Research has shown that the *METTL21C* gene plays a role in muscle growth and development in animals [58,59,60]. The *ACTC1* gene is linked to muscle cell development [49] and lipid metabolism [26] in animals. These TSGs represent promising biomarkers for marker-assisted selection. Taken together, this study provides unique insights for sheep breeding by identifying differentially expressed genes, muscle-specific genes, and novel regulatory networks related to meat quality traits, highlighting specific differences in the genetic regulation of WHC and tenderness. By incorporating these genes into genomic selection strategies, breeders can enhance the efficiency of selecting desirable meat quality traits, ultimately improving mutton production and consumer satisfaction. Nevertheless, the genetic mechanisms underlying the identified genes affecting meat quality remain unclear, and the small sample size may lead to the insufficient accuracy of the identified biomarkers. Therefore, further research with a larger sample size is warranted in the future.

## 5. Conclusions

By unraveling the transcriptomic networks underlying WHC and tenderness in Hu sheep, this research offers a solid foundation for comprehending the genetic basis of meat quality. The identified candidate genes (such as *SORBS1*, *FOXO1*, *CDH1*, *LEP*, *FABP4*, *PLIN1*, *CYLD*, and *METTL21C*) and pathways (including the lipid metabolic process, PPAR signaling pathway, cAMP signaling pathway, and ATP biosynthetic process) offer actionable targets for precision breeding programs. These advances hold significant potential for enhancing mutton quality, addressing global demands for sustainable and high-value meat production.

## Figures and Tables

**Table 1:** Primer pairs used for qRT-PCR.

Gene	Primer Names	Primer Sequences (5′–3′)	Annealing Temperature (°C)	Length (bp)
*NR4A3*	*NR4A3*-F	TAAATCCTGCCAGAGTTCCCT	52.6	168
	*NR4A3*-R	ACCTTATTATCCCTGGTGCTT		
*ARMC12*	*ARMC12*-F	ATAAGCTCCTTCACGGCAGA	50.5	104
	*ARMC12*-R	CCTTCAAAATCCAAGAGCCCAA		
*GREB1*	*GREB1*-F	GCTCCTCAGAAATGAATCAGC	50.8	127
	*GREB1*-R	ATTGACATTAACTCTTTGGCAT		
*NT5DC3*	*NT5DC3*-F	ACTTTCCTCCTAACATAGCCTT	51.5	146
	*NT5DC3*-R	GACCAATGCCTTCAAAGCAAC		
*UTS2R*	*UTS2R*-F	ACCCATTTCTCCCAACTGCCAT	58.5	127
	*UTS2R*-R	GGTCCTGCCTCCCTTGACACC		
*GRIN3A*	*GRIN3A*-F	TGTCCATCCTGACCACCGTTG	55.6	162
	*GRIN3A*-R	TAGTCTTGAAACGCTGTTGCT		
*EGR3*	*EGR3*-F	CAGCCACATTCAGTCATGCTC	52.5	100
	*EGR3*-R	TCTCTAGTGATCTTGCCAACCC		
*CPT1C*	*CPT1C*-F	GCAAATTCACCTGTTCGACGTT	56.4	122
	*CPT1C*-R	TGATCACGTCATCGCCCAT		
*ACTB*	*ACTB*-F	TCCGTGACATCAAGGAGAAGC	52–62	267
	*ACTB*-R	CCGTGTTGGCGTAGAGGT		

## Data Availability

Data will be made available on request from the corresponding author.

## Supplementary Materials

The following supporting information can be downloaded at: https://www.mdpi.com/article/10.3390/ani15111569/s1, Table S1: Quality of RNA and the number of sequenced reads. Table S2: List of DEGs for HWHC vs. LWHC comparison. Table S3: List of DEGs for HTN vs. LTN comparison.

## Abbreviations

The following abbreviations are used in this manuscript:

WHC	Water-holding capacity
DEGs	Differentially expressed genes
WGCNA	Weighted gene co-expression network analysis
LT	Longissimus thoracis
TPM	Transcripts per million
GO	Gene Ontology
KEGG	Kyoto Encyclopedia of Genes and Genomes
TSGs	Tissue-specific genes
